# The effect of comorbidity on health-related quality of life for injury patients in the first year following injury: comparison of three comorbidity adjustment approaches

**DOI:** 10.1186/1478-7954-9-10

**Published:** 2011-04-24

**Authors:** Juanita A Haagsma, Ed F van Beeck, Suzanne Polinder, Hidde Toet, Martien Panneman, Gouke J Bonsel

**Affiliations:** 1 Department of Public Health, Erasmus Medical Center, Erasmus University Rotterdam, P.O. Box 1738, 3000 CA, Rotterdam, The Netherlands; 2 Consumer Safety Institute, P.O. Box 75169, 1070 AD Amsterdam, The Netherlands

## Abstract

**Background:**

Three approaches exist to deal with the impact of comorbidity in burden of disease studies - the maximum limit approach, the additive approach, and the multiplicative approach. The aim of this study was to compare the three comorbidity approaches in patients with temporary injury consequences as well as comorbid chronic conditions with nontrivial health impacts.

**Methods:**

Disability weights were assessed using data from the EQ-5D instrument developed by the EuroQol Group and derived from a postal survey among 2,295 injury patients at 2.5 and 9 months after being treated at an emergency department. We compared the observed and predicted EQ-5D disability weights in comorbid cases using data from injury patients with and without comorbidity who were restored from their injuries at 9 months follow-up. The predicted disability weights were calculated using the maximum limit approach, additive approach, and multiplicative approach. The intraclass correlation coefficient (ICC) was used to test whether the values of the observed disability weights and the three model-predicted disability weights were correlated.

**Results:**

The EQ-5D disability weight of injury patients increased significantly with the number of comorbid diseases. The ICCs of the additive, multiplicative, and maximum limit models were 0.817, 0.778, and 0.674, respectively. Although the 95% confidence intervals of the ICCs of the three models overlap, the maximum limit model seems to fit less well than the additive and multiplicative models. For mild to moderate chronic disease (disability weight below 0.21), the association between predicted and observed disability weights was low.

**Conclusions:**

Comorbidity has a high impact on disability measured with EQ-5D. Ignoring the effect of comorbidity restricts the use of the burden of disease concept in multimorbid populations. Gains from health care or interventions may be easily overestimated if a substantial number of patients suffer from additional conditions. The results of this study found that in accounting for comorbidity effects, all three models showed a strong association between the predicted and observed morbid disability weight, though the maximum limit model seems to fit less well than the additive and multiplicative models. The three models do not fit well in the case of mild to moderate pre-existing disease.

## Background

Burden of disease studies quantify the health status of a population in order to facilitate the work of policymakers in setting priorities in health care and prevention [1,2]. Commonly, the outcome of such studies is expressed in disability-adjusted life years (DALYs), a summary measure of population health. Apart from the obvious advantages of a uniform summary measure of population health, the calculation and interpretation of the burden of disease in terms of DALYs can be complicated if multiple conditions co-exist in individuals. The fact that multiple conditions co-exist in individuals may be a matter of chance related to general susceptibility (e.g., advanced age) or the consequence of a disease with multiple systemic manifestations and remote complications. Nevertheless, assignment of the observed burden to separate conditions, either in descriptive terms or in terms of the total computed burden of disease, is arbitrary, and several difficulties emerge.

The first difficulty is that the straightforward additive use of DALYs per disease is limited as this assumes that the total burden of two or more diseases is the sum of the burden of diseases taken separately. A second difficulty occurs in so-called counterfactual impact analysis of risk factors. What happens in terms of population DALYs if one disease is eradicated? The answer depends on a valid solution of the comorbidity assignment problem.

Furthermore, the comparative outcomes research faces problems to the extent that outcome differences can virtually disappear through the overriding effects of comorbid conditions. The common practice to exclude patients with comorbidity from participation in trials postpones rather than solves the question concerning the average population effect of an intervention.

Comorbidity is defined as the presence of any clinical condition that qualifies for formal classification as a disease additional to the disease under study. Risk factors such as advanced age, ethnic background, or obesity are essentially not comorbid conditions, although the principles described below also might be applicable.

At present, three approaches exist to deal with the impact of comorbidity with regard to burden of disease studies [3,4]. These approaches are elaborated here.

The first approach is the maximum limit approach. This approach counts the disease with the highest overall disability weight. The approach assumes that a comorbid disease does not affect disability of a patient with a primary disease, unless the comorbid disease, in general terms, exceeds the disability of the former.

The second approach is the additive approach. This approach assumes that the additional effect, or more precisely, the utility loss, of comorbid disease simply adds to the effect (utility loss) of the primary disease observed in uniconditional patients. The disability weights of the comorbid diseases are added up.

The third approach is the multiplicative approach. This method assumes that a comorbid disease increases the utility loss of a patient, though it is less than the sum of the utility loss of both diseases independently.

Here, we present a systematic comparison of the three comorbidity adjustment approaches in patients with injuries and common diseases with non-trivial health impacts as the secondary condition.

## Methods

### Design

**Figure 1:**
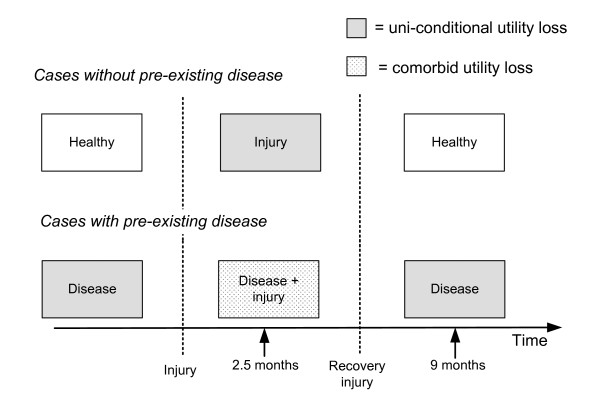
**Design of the study**.

We compared the observed (gold standard) and predicted disability weights in comorbid cases, using data from the EQ-5D instrument developed by the EuroQol Group for injury patients with and without comorbidity. For this comparison, long-term follow-up data from injury patients could be used because the onset of injury is acute, causing immediate, yet usually temporary functional loss. This allows the measurement of utility loss due to comorbid injury and disease (comorbid utility loss) and the measurement of utility loss due to the injury and disease separately (uniconditional utility loss). The comorbid utility loss was used to calculate the observed disability weights in comorbid cases. The uniconditional utility loss was used to calculate the predicted disability weights with the maximum limit, additive, and multiplicative approaches. See Figure 1 for a schematic model of the design.

### Patient data

The primary data sources were existing national registry data on injured patients upon hospital admission, enhanced with functional outcome data obtained from patients by surveys at regular intervals.

#### Registry data

The registry data were derived from the Dutch Injury Surveillance system, a permanent registry of injuries treated at the emergency departments (ED) of a representative sample of 17 hospitals (about 10%-15% of ED capacity) in the Netherlands. The registry collects information on age and sex of the patient, cause and type of the injury, body region affected, and treatment of the sustained injury [5].

#### Follow-up survey

The follow-up survey was administered between 8 October 2001 and 31 December 2002 to a sample of 8,564 patients aged 15 years and older who were treated at the ED and whose registry data were available through the Dutch Injury Surveillance System [5]. The patients were treated at the ED followed by either hospital admission or direct discharge to the home environment. The sample of patients consisted of victims of traffic, home and leisure, occupational, and sport accidents. The sustained injuries varied from minor to severe injuries and hospitalized and nonhospitalized patients. The sample of patients was stratified, oversampling hospitalized patients. Each injury patient of the selected sample received a postal questionnaire 21/2 months after the injury, and 3,167 (37%) responded. The first questionnaire was anonymous for privacy reasons. At 5, 9, and 24 months following the injury, a follow-up questionnaire was sent to patients who responded to the preceding questionnaire. For these questionnaires, the patients needed to give permission by an informed consent form. The present study used a sample of 2,295 respondents (i.e., 27% of the original sample) who responded to the 21/2-month and 9-month post-trauma survey.

### Utility measurement and disability weight

To measure utility after injury, the questionnaire included the multi-utility attribute instrument EQ-5D. With the EQ-5D classification system, subjects describe their health state by assigning themselves to one of three function levels (grades) in five separate domains: mobility, self-care, usual activities, pain/discomfort, and anxiety/depression [6,7]. Subsequently, the utility weight of that health state was computed by a formula that first yields a partial weight score for each domain depending on the reported level, and then adds the utility weights (also referred to as the tariff) derived at an earlier stage from preference data of the United Kingdom population [8].

For the subsequent calculation of the EQ-5D disability weights, we used the population health index of the population of the United Kingdom, adjusted for age and sex [9]. To calculate the EQ-5D disability weight, the measured EQ-5D utility weight was subtracted from the UK population norm for a person of that age and sex (see additional file 1). For instance, 21/2 months after sustaining a fracture of the lower extremity, a male patient aged 30 reported some problems with walking and performing usual activities, as well as moderate pain or discomfort (EQ-5D profile 21221), resulting in a utility weight of 0.69. The UK population norm for a male aged between 25 and 35 is 0.93. The EQ-5D disability weight was subsequently calculated by subtracting 0.69 from 0.93, resulting in an EQ-5D disability weight of 0.24. Patients with a disability weight of ≤ 0 were excluded from the analysis.

### Observed disability weight in comorbid cases

The survey included a question that asked whether patients were restored from their injuries (yes/no). This study was restricted to injury patients who indicated that they were restored from their injury after nine months of follow-up. Furthermore, the questionnaire included 19 items regarding the presence of one or more chronic diseases prior to the injury to assess comorbidity [10]. Comorbidity is defined as the presence of any co-existing medical diseases or disease processes additional to the injury that the injury patients sustained [11]. We selected six persisting diseases or disease symptoms that were most often reported - chronic, non-specific lung disease; heart disease; diabetes; backache; osteoarthritis; and rheumatoid arthritis.

The comorbid injury and disease disability weight was obtained from the 21/2 month EQ-5D data reported by injury patients with comorbid disease.

### Predicted disability weight in comorbid cases

To predict the disability weight in comorbid cases with the maximum limit, additive, and multiplicative approaches, uniconditional disability weights were used. The uniconditional disability weight of the injury was obtained from the 21/2-month EQ-5D data reported by injury patients without comorbid disease. The effects of the injury consequences on utility can be measured at 21/2 months after sustaining the injury, given that the selected patients were restored at 9 months post-injury. This assumption allows measurement of the disability effects of the comorbid disease only. Therefore, the uniconditional disability weights of the disease were obtained from the 9-month EQ-5D data of injury patients with comorbid disease.

These uniconditional disability weights were then used to calculate the predicted disability weight in cases of injury and comorbid disease according to the three approaches. In the examples supporting the formulas used to calculate the comorbid disability weights, the injury type "leg fracture" serves as the primary disease, and heart disease is an example of pre-existing disease.

#### Predicted disability weights: maximum limit approach

To calculate comorbid disability weight with the maximum limit approach, we used the following formula:

The maximum limit approach is biased if two conditions affect different health domains of the EQ-5D (underestimation by model-based cancellation).

#### Predicted disability weights: additive approach

To calculate comorbid disability weight with the additive approach, we used the following formula:

One limitation is that the combined disability may exceed 1.0. Bias may arise if both diseases affect the same health domain of the EQ-5D; for example, the effect of diabetic foot amputation does not add to a lower leg fracture of the same leg (overestimation by data cancellation).

#### Predicted disability weights: multiplicative approach

To calculate comorbid disability weight with the multiplicative approach, we used the following formula:

### Analysis

For the analysis of the data, the Statistical Package for the Social Sciences (SPSS) version 16.0 was used (SPSS Inc., Chicago, IL). One-way ANOVA was used to test for differences in disability weights among patients with and without comorbid disease. The intraclass correlation coefficient (ICC) was used to determine the goodness-of-fit of the predicted comorbid disability weights using the three comorbidity adjustment methods. In this way, we tested whether the values of the observed disability weights and the three model-predicted disability weights were correlated.

To test whether the relations between observed and predicted disability weights depended on the severity of the comorbid disease, the pre-existing disease was grouped into two severity classes using the median value of the 9-month disability weight of patients with comorbid disease (0.21) as a cut-off (range 0.0-0.21 is the less severe group).

## Results

Of the 2,295 injury patients who completed the follow-up surveys 21/2 and 9 months after they were treated at the ED, 1,438 (62.7%) indicated they were restored from their injury at 9 months follow-up.

The 21/2-month EQ-5D disability weights increased significantly with the number of comorbid diseases (F = 31.8, p < 0.001). For instance, injury patients with a concussion and no comorbid disease had a mean EQ-5D disability weight of 0.04, whereas similar patients with two or more comorbid diseases had mean EQ-5D disability weights of 0.08 and 0.35, respectively. Table 1 shows the uniconditional disability weights of the injury groups that were obtained from 21/2-month EQ-5D data of injury patients without comorbidity.

**Table 1:** Mean uniconditional disability weights of the injury groups, obtained from EQ-5D data from injury patients without comorbid disease at 21/2 months follow-up.

Injury type	N	**Mean**^1^	**CI**^2^
Skull - brain injury	192	.06	.04-.08
Facial fracture, eye injury	51	.05	.02-.07
Spine, vertebrae	26	.18	.10-.27
Internal organ injury	61	.05	.03-.07
Upper extremity fracture	188	.07	.05-.09
Upper extremity, other injury	64	.09	.05-.12
Hip fracture	12	.21	.11-.32
Lower extremity fracture	151	.15	.13-.18
Lower extremity, other injury	74	.13	.10-.16
Superficial injury, open wounds	158	.06	.04-.07
Burns	14	.05	<.01-.12
Poisonings	16	.06	<.01-.12
Other injury	51	.06	.04-.09

^1^Adjusted for age and sex of the patient

^2 ^CI = confidence interval

Table 2 presents the mean uniconditional EQ-5D disability weights of the diseases. Heart disease had the lowest mean EQ-5D disability weight of 0.07. Osteoarthritis had the highest mean disability weight (0.38).

**Table 2:** Mean uniconditional disability weights of pre-existing diseases, obtained from EQ-5D data of injury patients at 9 months follow-up

Pre-existing disease	n	**Mean**^1^	**CI**^2^
CNLD^3^	7	.12	.05-.19
Heart disease	11	.07	.07-.15
Diabetes	6	.21	.01-.51
Backache	8	.26	.01-.69
Osteoarthritis	30	.38	.20-.56
Rheumatoid arthritis	10	.16	.01-.41
Other disease	51	.23	.11-.19

^1 ^Adjusted for age and sex of the patient

^2 ^CI = confidence interval

^3 ^CNLD = Chronic nonspecific lung disease

The mean observed and predicted comorbid disability weights, where the predicted disability weights were calculated with the three different adjustment approaches, are shown in Table 3.

**Table 3:** Mean observed and predicted comorbid disability weights^1 ^(pre-existing diseases and injury)

		Predicted		
		
	Observed	*Max limit approach*	*Additive approach*	*Multiplicative approach*
CNLD^2 ^and injury	0.23 (0.04)	0.18 (0.02)	0.30 (0.06)	0.28 (0.05)
Heart disease and injury	0.28 (0.26)	0.17 (0.01)	0.26 (0.08)	0.24 (0.07)
Diabetes and injury	0.33 (0.17)	0.18 (0.02)	0.30 (0.01)	0.28 (0.01)
Backache and injury	0.19 (0.19)	0.12 (0.02)	0.14 (0.02)	0.14 (0.02)
Osteoarthritis and injury	0.52 (0.35)	0.39 (0.29)	0.49 (0.34)	0.44 (0.28)
Rheumatoid arthritis and injury	0.25 (0.19)	0.18 (0.04)	0.25 (0.08)	0.23 (0.07)
Other disease and injury	0.30 (0.22)	0.22 (0.13)	0.30 (0.17)	0.28 (0.14)

^1 ^Mean disability weight (standard deviation)

^2 ^CNLD = Chronic nonspecific lung disease

**Figure 2:**
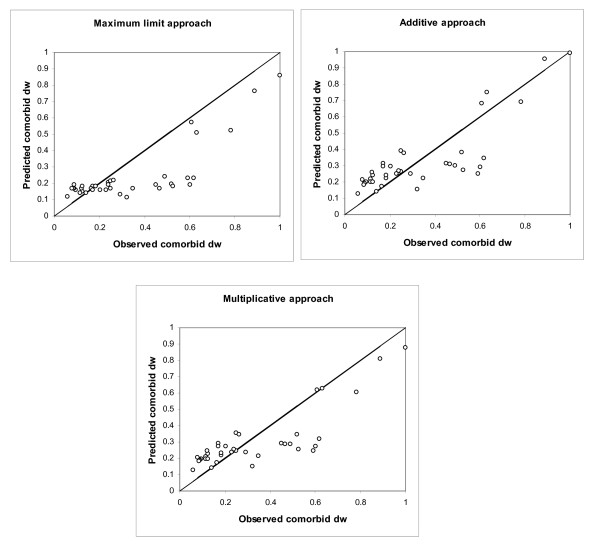
**Observed and predicted disability weights for comorbid conditions**.

Figure 2 shows the relationship between the observed and predicted disability weights for the injury types and comorbid diseases. The predicted disability weights were calculated for each injury patient separately. In Figure 2, each data point represents a patient.

The ICC between the observed and predicted comorbid disability weights was highest for the additive approach (ICC = 0.817; 95% confidence interval [CI]: 0.681-0.898). The ICCs of the multiplicative approach and maximum limit approach were 0.778 (95% CI: 0.463-0.812) and 0.674 (95% CI: 0.619-0.876), respectively.

As shown in Table 4, in cases of severe chronic disease, all three models showed a strong association of the predicted and observed comorbid disability weight. For mild to moderate chronic disease (disability weight below 0.21), the association between predicted and observed disability weights was low, especially for the maximum limit model.

**Table 4:** Goodness-of-fit of observed comorbid disability weights and comorbid disability weights predicted with the three adjustment approaches, stratified by severity of the disease

Severity	Maximum limit approach ICC (95% CI)	Additive approach ICC (95% CI)	Multiplicative approach ICC (95% CI)
Mild to moderate chronic disease (dw < 0.21)	.073 (-.269 - .401)	.399 (.073 - .649)	.351 (0.17 - .615)
Severe chronic disease (dw ≥ 0.21)	.887 (.530 - .979)	.953 (.780 - .992)	.933 (.699 - .988)

## Discussion

The results of this study show that the EQ-5D disability weight of injury patients increases with the number of comorbid diseases. The goodness-of-fit of the three models to account for comorbidity effects was found to be high. Although the 95% confidence intervals of the ICCs of the three models overlap, the maximum limit model seems to fit less well than the additive and multiplicative models.

Analysis of the goodness-of-fit of the three models stratified by severity of the pre-existing disease indicated that the three models do not fit well in cases of mild to moderate pre-existing disease.

Very few studies have used actual patient data to verify the validity of comorbidity adjustment approaches. Flanagan et al. tested the multiplicative approach using empirical utility data from the Canadian Community Health Survey [12]. They showed that observed and predicted utility was highly associated. However, it should be noted that Flanagan et al. tested only the multiplicative approach and that they measured health loss using the Health Utilities Index (HUI), whereas in the current study, the EQ-5D was used. Both are generic instruments. The functional health state of the patient and the utility weights derived from the population are based on generic attributes and without regard to the underlying disease, disease-specific key symptoms, prognosis, or treatment. Evidence suggests that the sensitivity of the EQ-5D is low compared to the HUI, implying that the EQ-5D does not measure disability where the HUI does [13]. Additionally, Polinder et al. showed that among injury patients, the HUI is more sensitive for comorbid disease compared to the EQ-5D [14]. This might be caused by the crudeness of the levels, three in the case of the EQ-5D compared to five or six levels of the HUI. Comparison of the visual analogue scale (VAS) scores of patients with and without comorbid disease might clarify this issue. The VAS valuation technique requires patients to score their health state on a vertical thermometer graded from 0 (worst possible health state) to 100 (best possible health state). Unfortunately, it was not possible to assess the effects of comorbid disease and injury with the VAS because too many VAS scores were missing.

An important limitation that applies to the current study and the study of Flanagan et al.[12] is that utility scores were used to test the comorbidity adjustment approaches rather than the impact on the separate health domains of the multi-attribute utility instruments. This limitation may be overcome by a fourth adjustment approach that starts from the domain- specific impact of a disease without comorbidity and compares this impact to the estimated domain impact of the comorbidity only. By selecting the maximum impact for each domain, a maximum limit profile is derived with the worst of both diseases for each domain. Subsequently, the total utility and disability weight of the maximum profile are conventionally calculated. This approach can accommodate co-existing diseases that share affected domains or the presence of two or more comorbid diseases. It does, however, require detailed descriptive data.

A second limitation that may have affected the results of the current study is that health loss of co-existing disease and temporary consequences of injury were measured using patient-reported EQ-5D data. As a result, adaptation might have affected the patient-reported EQ-5D data. The selected co-existing diseases were chronic, and adaptation to their chronic health state might have caused patients to value their health state as less severe. This effect is especially found regarding chronically ill patients [15,16]. The level of adaptation possibly differs between patients with mild to moderate and severe chronic disease and this may explain the differences found in association between predicted and observed disability weights in cases of severe chronic disease compared to mild to moderate chronic disease.

Of the injury patients restored from their injuries 9 months after injury, 12% reported comorbid disease. This percentage was lower compared to the total sample of injury patients, of which 29% of the patients reported comorbid conditions. This latter percentage is comparable to the proportion of patients with comorbid disease found in previous studies [17,18].

A third limitation of the current study is that pre-injury utility scores of the injury patients were unavailable. Therefore, 9-month disability weights were used to calculate the comorbid disease with the three comorbidity adjustment approaches. We assumed that the 9-month disability weights of patients who were restored from their injury capture the health loss due to chronic, pre-existing disease without the effects of the injury and that the health loss caused by the pre-existing comorbid disease at 9 months was similar to the health loss at 21/2 months post-injury. However, the severity of the disease might have changed over time. Regarding rheumatioid arthritis, for instance, symptoms may vary over time. This may have resulted in either under- or overestimation of the health loss due to the pre-existing disease at 21/2 months post-injury.

Furthermore, the comorbidity adjustment approaches were tested with EQ-5D data. The use of the EQ-5D to assess the impact of injury and comorbidity has its limitations. One of these limitations is that the EQ-5D has large ceiling effects in the general population [19]. These ceiling effects may also affect the measurement of health-related quality of life measured by EQ-5D among injury patients with mild short-term injury. However, in this study, in order to use empirical data to test the comorbidity adjustment approaches, actual reported patient data were required. Therefore, we have used disability weights based on EQ-5D data. Evidence suggests that EQ-5D utility scores of individuals may be influenced by medical as well as nonmedical factors, such as age and sex, educational level, and marital status [9,20]. In the current study, the disability weights were adjusted for age and sex. Other nonmedical factors that may have affected the reported EQ-5D health states reported by the injury patients were not taken into account.

## Conclusions

By assuming a single-disease hypothesis, the standard application of the DALY metric fails to consider the fact that more than one disease may exist simultaneously in a patient [21]. Apart from the difficulty of distinguishing primary and secondary diagnosis, ignoring comorbid disease in burden of disease estimates restricts the use of the DALY in multimorbid populations, such as the elderly in high-income countries. Gains from care may easily be overestimated if a substantial number of patients suffer from additional diseases. In cases where disorders are more often held to be secondary than primary, their significance in burden of disease studies and the benefit of eradication may be underestimated. This has implications for priority setting and prevention and may lead to wrongful policy recommendations.

The results of this study showed that the goodness-of-fit of available comorbidity adjustment approaches was high. The maximum limit model, however, seems to fit less well than the additive and multiplicative models. Furthermore, analysis of the goodness-of-fit of the three models stratified by severity of the pre-existing disease indicated that the three models do not fit well in cases of mild to moderate pre-existing disease.

To improve current approaches to adjust for comorbidity, we recommend more research on the effects of comorbidity on health-related quality of life. In this study, the effects of temporary injuries and chronic disease have been investigated. It remains to be investigated whether the effect of multiple chronic diseases on health-related quality of life and the performance of the available adjustment approach are similar.

In addition, other adjustment approaches should be developed. The currently available methods rely upon the disability weight, yet other methods, such as a domain-specific method, should be explored.

## List of abbreviations

CI: Confidence interval; DALY: Disability-adjusted life year; ED: Emergency department: ICC: Intra-class correlation; VAS: Visual analogue scale

## Competing interests

The authors declare that they have no competing interests.

## Authors' contributions

JH executed the statistical analysis and drafted the manuscript. EvB supervised the statistical analysis. SP participated in the design and execution of the panel study. HT collected patient survey data necessary for analysis and assisted with the drafting of the manuscript. MP collected patient survey data necessary for analysis and assisted with the drafting of the manuscript. GB supervised and participated in the design of the study and the drafting of the manuscript. All authors read and approved the final manuscript.

## Supplementary Material

Additional file 1 **EQ-5D UK population norms per age and sex category**. EQ-5D UK population norms per age and sex category which were used for the calculation of EQ-5D disability weights. Click here for file
