# Supplementary material for: The effect of comorbidity on health-related quality of life for injury patients in the first year following injury: comparison of three comorbidity adjustment approaches
Source: Popul Health Metr. 2011 Apr 24;9:10. doi: 10.1186/1478-7954-9-10 (PMC3096905; doi:10.1186/1478-7954-9-10)
Supplement: Additional file 1 — EQ-5D UK population norms per age and sex category. EQ-5D UK population norms per age and sex category which were used for the calculation of EQ-5D disability weights. [file 1478-7954-9-10-S1.DOC]

**Supplementary table**

EQ-5D UK population norms per age and sex category. From Kind P, Hardman G, Macran S: UK population norms for EQ-5D. York: Centre of Health Economics, University of York; 1999.

Table 1. EQ-5D UK population norms per age and sex category.

|  | Males | Females | All |
| --- | --- | --- | --- |
| All | 0.86 | 0.85 | 0.86 |
|  |  |  |  |
| <25 years | 0.94 | 0.94 | 0.94 |
| 25-34 years | 0.93 | 0.93 | 0.93 |
| 35-44 years | 0.91 | 0.91 | 0.91 |
| 45-54 years | 0.85 | 0.84 | 0.85 |
| 55-64 years | 0.80 | 0.78 | 0.81 |
| 65-74 years | 0.78 | 0.78 | 0.78 |
| 75+ years | 0.73 | 0.71 | 0.75 |
